# Influence of Electrostatic Interactions on the Self-Assembly of Charged Peptides

**DOI:** 10.3390/gels11010080

**Published:** 2025-01-20

**Authors:** Xue Sun, Bolan Wu, Na Li, Bo Liu, Shijun Li, Liang Ma, Hangyu Zhang

**Affiliations:** 1 Faculty of Medicine, Dalian University of Technology, Dalian 116033, China; 2 Liaoning Key Lab of Integrated Circuit and Biomedical Electronic System, School of Biomedical Engineering, Dalian University of Technology, Dalian 116024, China; 2251740842@mail.dlut.edu.cn (B.W.); lina316@dlut.edu.cn (N.L.); lbo@dlut.edu.cn (B.L.)

**Keywords:** self-assembling peptide, electrostatic interactions, 3D printing, hydrogel, self-healing

## Abstract

Peptides can be designed to self-assemble into predefined supramolecular nanostructures, which are then employed as biomaterials in a range of applications, including tissue engineering, drug delivery, and vaccination. However, current self-assembling peptide (SAP) hydrogels exhibit inadequate self-healing capacities and necessitate the use of sophisticated printing apparatus, rendering them unsuitable for 3D printing under physiological conditions. Here, we report a precisely designed charged peptide, Z5, with the object of investigating the impact of electrostatic interactions on the self-assembly and the rheological properties of the resulting hydrogels. This peptide displays salt-triggered self-assembly resulting in the formation of a nanofiber network with a high β-sheet content. The peptide self-assembly and the hydrogel properties can be modified according to the ionic environment. It is noteworthy that the Z5 hydrogel in normal saline (NS) shows exceptional self-healing properties, demonstrating the ability to recover its initial strength in seconds after the removal of shear force, thus rendering it an acceptable material for printing. In contrast, the strong salt shielding effect and the ionic cross-linking of Z5 hydrogels in PBS result in the bundling of peptide nanofibers, which impedes the recovery of the initial strength post-destruction. Furthermore, incorporating materials with varied charging properties into Z5 hydrogels can alter the electrostatic interactions among peptide nanofibers, further modulating the rheological properties and the printability of SAP hydrogels.

## 1. Introduction

Hydrogels are an important class of biomaterials consisting of hydrophilic polymers, which are widely used in a variety of biomedical applications, such as cell culture, tissue engineering, drug delivery platforms, cancer therapy, and regenerative medicine, due to their high water content, biocompatibility, tunable mechanical strength, and porous structure [1,2,3]. They can be synthesized either from natural and synthetic high molecular polymers or through the self-assembly of small molecules to form physically cross-linked networks. The latter approach is particularly advantageous due to the smaller size of the gelator and the reversibility or responsiveness of the resultant hydrogels, stemming from dynamic intermolecular non-covalent interactions. SAP hydrogels are synthesized from naturally occurring amino acids and form rich non-covalent interactions. These SAPs are sensitive to environmental stimuli, such as temperature, pH, ion concentration, and light [4,5,6,7,8,9,10,11,12,13,14]. In particular, self-assembling peptides containing alternating hydrophilic and hydrophobic amino acids have been the subject of much pioneering work in many applications [15,16,17]. Critical to the molecular self-assembly is control over the functionalization of the monomeric structural units [18]. The encoded amino acid sequences in peptides play a crucial role in defining conformation, which, along with chemistry [19,20], determines the structure of the self-assembled entities. Consequently, these factors collectively influence the overall mechanical properties of the SAP-based hydrogels, with the design of the amino acid sequence being key in regulating their macroscopic properties.

SAP-based hydrogels are primarily governed by a variety of non-covalent interactions, such as electrostatic interactions [21,22], hydrophobic interactions [23,24,25,26,27], hydrogen bonds [28,29,30,31], and π-π stacking [32,33,34,35]. Among them, hydrophobic interactions and hydrogen bonding positively influence peptide self-assembly, whereas electrostatic interactions play a more complex role, including both electrostatic repulsion and attraction. In the case of charged peptide molecules, electrostatic repulsion can inhibit the assembly. The fine-tuning of the balance between electrostatic and other non-covalent interactions allows for precise control over peptide self-assembly, thereby influencing the mechanical properties of the resultant peptide hydrogels.

Electrostatic interactions in peptide hydrogels can be modulated through three primary approaches. One of these approaches involves designing the amino acid sequence of the peptide. The number and distribution of positively and/or negatively charged amino acids significantly impact the assembly. For example, Suvrat Chowdhary et al. designed an amphiphilic peptide with a large number of fluorinated amino acids [36]. The electronegative fluorine atoms in the peptide form electrostatic attractions with positively charged atoms on the main and side chains, illustrating stable self-assembly predominantly influenced by electrostatic interactions. Secondly, the ionic concentration in the environment is another key factor. In environments with multivalent counterions, peptide nanofibers form a dense network via ionic cross-linking. Conversely, in the absence of multivalent counterions, controlling the concentration of environmental monovalent ions can significantly impact peptide self-assembly [37,38,39]. Another approach involves incorporating charged materials to create composite hydrogels, thereby influencing the assembly structure and the mechanical properties of the hydrogel. Shy et al. demonstrated that mixing peptide solutions with opposing charges yielded a novel SAP hydrogel [22]. However, current SAP hydrogels have poor self-healing properties and require complex printing devices, making them unsuitable for 3D bioprinting under physiological conditions.

Here, we developed a SAP hydrogel with excellent self-healing properties and the capability of 3D printing under physiological conditions. Peptide Z5 exhibits salt-triggered self-assembly to form a nanofiber network that is rich in β-sheet structure. By varying the ionic environment, the internal electrostatic interactions among nanofibers can be tuned, which further influence the rheological properties of the SAP hydrogels. Interestingly, Z5 hydrogel in normal saline (NS) displays exceptional self-healing properties as it can recover its initial strength seconds after releasing the shear force, thereby rendering it acceptable for printing applications. The printing quality can be further improved by mixing positively charged gelatin with Z5, which regulates the electrostatic interactions among nanofibers.

## 2. Results and Discussion

### 2.1. Salt-Triggered Peptide Self-Assembly

Peptide Z5, with the sequence Ac-KVQVKVQVKVQV-COOH, carrying two units of positive charge under physiological conditions, was developed to study the influence of electrostatic interactions on peptide self-assembly and the rheological properties of the resulting hydrogels. Lysines are included in the sequence and are distributed separately to allow for mild intermolecular electrostatic repulsion. The hydrophobic side chain of valine, which is thought to have a higher propensity to form β-sheet structures, provides hydrophobic interactions that promote the self-assembly of the peptide [40]. Glutamine, a hydrophilic neutral amino acid, is incorporated to provide richer hydrogen bonding for self-assembly.

**Figure 1:**
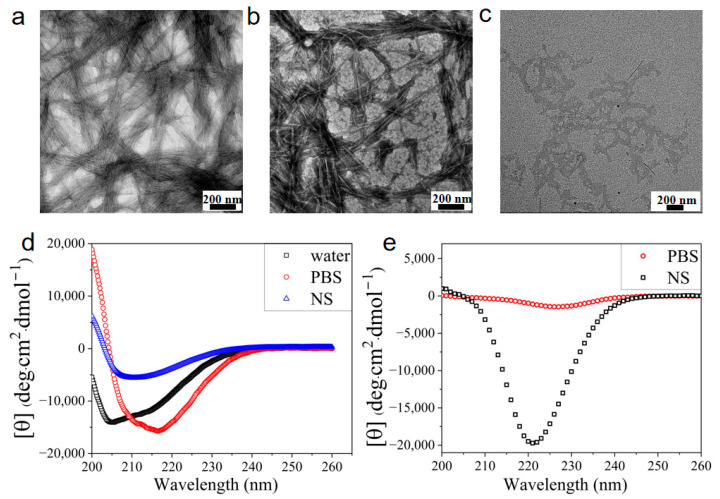
Salt-triggered self-assembly of Z5. TEM images of 100 μM Z5 in PBS (**a**), in NS (**b**), and in DI water (**c**). (**d**) CD spectra of 100 μM Z5 in DI water, PBS, and NS obtained in a CD cuvette with a 1 mm path length. (**e**) CD spectra of 7 mM Z5 hydrogels in PBS and NS obtained in a CD cuvette with a 0.1 mm path length.

**Figure 2:**
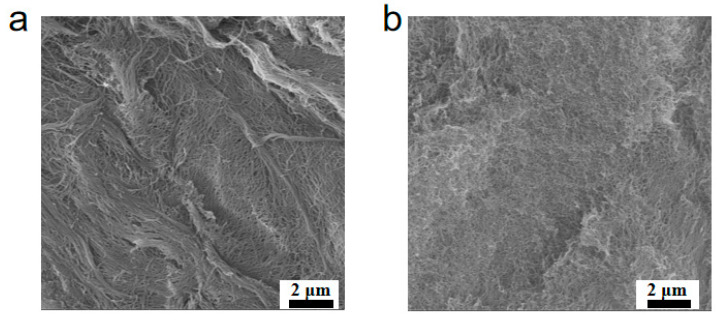
SEM images of Z5 hydrogels (2 wt%) in PBS (**a**) and in NS (**b**).

Salt-triggered self-assembly of the charged peptide Z5 is observed with transmission electron microscopy (TEM) and circular dichroism (CD) spectroscopy. TEM images of Z5 solutions at a low concentration (100 μM) revealed the presence of densely distributed nanofiber bundles in PBS (Figure 1a) and the formation of entangled individual nanofibers devoid of bundling in NS (Figure 1b). In contrast, nanofibers were not found in the Z5 solution in the absence of salt (Figure 1c). The salt shielding effect in PBS or NS markedly attenuates the electrostatic repulsion between the charged peptide molecules, thereby triggering self-association promoted by hydrophobic interactions and hydrogen bonding. CD spectra reveal the conformational transition from a mixed secondary structure for Z5 at 100 μM in water to a β-sheet, characterized by a negative peak at around 215 nm upon the introduction of PBS or NS (Figure 1d). It worth noting that the characteristic β-sheet peak shifts to larger than 220 nm in the CD spectra of Z5 hydrogels at 1 wt% (Figure 1e), probably due to twisting of the β-sheet structure at high concentrations. The notable reduction in the CD signal of the Z5 hydrogel in PBS may be attributed to nanofiber coagulation, as the nanofiber network would be disrupted when placing the brittle hydrogel in the CD cuvette with a 0.1 mm path length. In order to observe the intricate structure of Z5 hydrogels, scanning electron microscopy (SEM) was performed. In PBS, owing to ionic cross-linking and the salt shielding effect, the hydrogel forms a dense network of nanofibers, which further coalesce into bundles, observable as tens to hundreds of nanometer-thick fiber bundles in the SEM image (Figure 2a). In contrast, the positively charged nanofibers are closely woven into a dense network without bundling in NS (Figure 2b), resulting from the absence of polyvalent anion-induced cross-linking.

**Figure 3:**
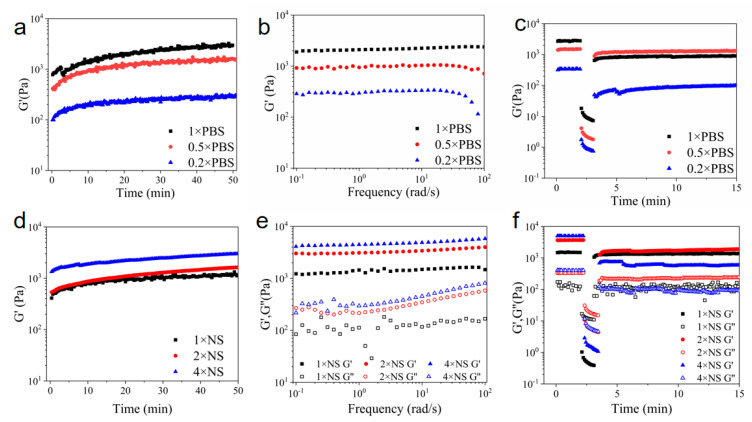
Rheological properties of 2 wt% Z5 hydrogels. Time sweeps of Z5 hydrogels in 0.2×, 0.5×, and 1× PBS (**a**) and in 1×, 2×, and 4× NS (**d**) monitoring the gelation process. Frequency sweeps of Z5 hydrogels in 0.2×, 0.5×, and 1× PBS (**b**) and in 1×, 2×, and 4× NS (**e**). Time sweeps with alternating low–high strains of Z5 hydrogels in 0.2×, 0.5×, and 1× PBS (**c**) and in 1×, 2×, and 4× NaCl (**f**) for the evaluation of self-healing properties.

Initially, the ionic environment in the solution was varied to ascertain the impact of electrostatic interactions on the hydrogel properties. Rheological tests were conducted on Z5 hydrogels (2 wt%) in PBS and normal saline (Figure 3). Upon the introduction of ions, the storage modulus (G′) of Z5 exhibited a rapid increase, indicating immediate gelation (Figure 3a,d). The rheological results demonstrate that the storage modulus (G′) and loss modulus (G″) roughly remained constant over the entire frequency range in the selected solution environments (Figure 3b,e).

In phosphate buffers, polyvalent anions, such as phosphate and hydrogen phosphate, attract the positively charged peptide molecules, thereby facilitating cross-linking among nanofibers. As the concentration of PBS decreases, cross-linking is attenuated, resulting in a thinner nanofiber network and a concomitant reduction in G′. Moreover, the Z5 hydrogel in 0.5× PBS displayed liquid-like behavior under high strain, followed by rapid recovery of the initial G′ after releasing the high strain (Figure 3c), in contrast to those in 0.2× and 1× PBS, which exhibited brittleness. In 1× PBS, the strong salt shielding effect and the ionic cross-linking resulted in the bundling of peptide nanofibers, which impeded recovery of the initial G′ post-destruction. In 0.5× PBS, the reduced ion concentration diminished the salt shielding effect and amplified the electrostatic repulsion among the positively charged peptide nanofibers, thereby diminishing the bundling ability and allowing the hydrogel to revert to its original G′ within a minute of destruction. Lastly, in 0.2× PBS, the low ion concentration resulted in inadequate salt shielding and self-assembly. In this case, the interconnection among the peptide nanofibers in the weak hydrogel was insufficient to support restoration of the initial G′ once disrupted.

In NaCl environments, the hydrogel network is primarily maintained through physical entanglement, as there is an absence of ionic cross-linking among nanofibers in Z5 hydrogels due to the presence of only monovalent ions. Z5 hydrogels (2 wt% and 1 wt%) in normal saline (NS) also exhibit shear-thinning and rapid recovery behavior, similar to that observed in 0.5× PBS (Figure S1c). The increase in monovalent ion concentration in NS, compared to that in 0.5× PBS, amplified the salt shielding effect, which is speculated to effectively compensate for the absence of ionic cross-linking. This caused the nanofibers to interact with each other and enhanced entanglement, thus rendering G′ comparable to that observed in 0.5× PBS. Notably, at a concentration of 1 wt% (Z5), gelation was comparatively slower than at a concentration of 2 wt%, resulting in a prolonged duration to achieve the peak G′ and a weaker network (Figure S1a,b), and consequently, a relatively longer recovery period post-destruction (Figure S1c). As the concentration of NaCl increased, Z5 nanofibers tended to bundle, which impeded the recovery of the initial G′ post-destruction (Figure 3f), probably due to the intensified salt shielding effect, which leads to a denser nanofiber network with increased G′. Accordingly, the Z5 hydrogels were unable to recuperate following the disruption of weak interactions caused by high strain, exhibiting brittleness in 2× and 4× NS.

### 2.2. Co-Assembly of Charged Peptides

**Figure 4:**
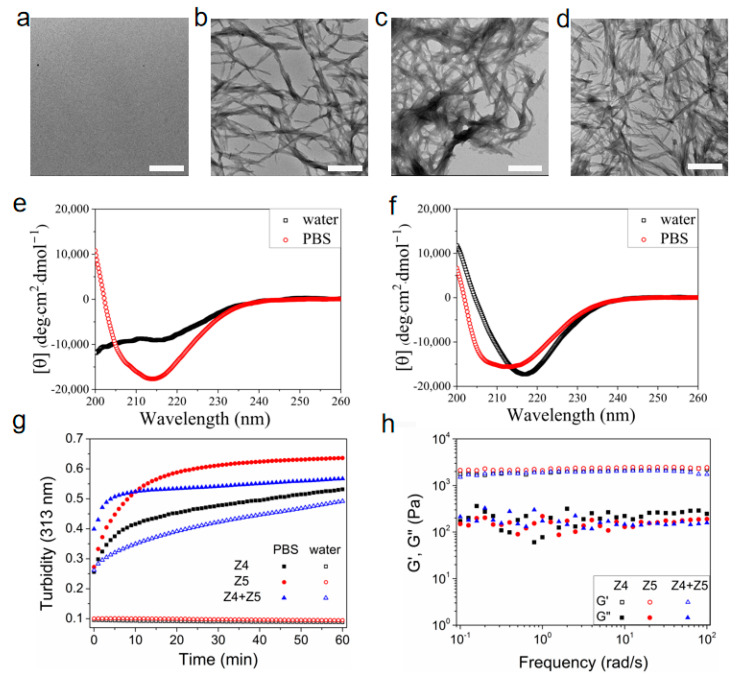
The co-assembly of Z5 and Z4. TEM images of 100 μM Z4 in DI water (**a**) and in PBS (**b**). TEM images of peptide mixture solutions with 50 μM Z5 and 50 μM Z4 in DI water (**c**) and in PBS (**d**). Scale bar: 500 nm. CD spectra of 100 μM Z4 in DI water and in PBS (**e**). CD spectra of peptide mixture solutions with 50 μM Z5 and 50 μM Z4 in DI water and in PBS (**f**). Turbidity test of peptide solutions or hydrogels (**g**). Rheologicdal frequency sweeps of peptide hydrogels (**h**).

A negatively charged peptide, designated Z4, with the sequence NH_2_-QVEVQVEVQVEV-Am, was introduced to Z5 for co-assembly through electrostatic interactions. The charge-complementary amino acid sequences in Z4 and Z5 grant co-assembly regardless the ionic environment. Z5 and Z4 each self-assemble in PBS to form nanofiber structures (Figure 1a,c and Figure 4a,b) at a low concentration (100 μM). Furthermore, the salt-triggered self-assembly is also corroborated by CD spectra, which exhibit the β-sheet peak at approximately 216 nm and a minor shoulder at around 206 nm (Figure 1d and Figure 4e), indicative of the presence of disordered structures. The CD spectra of Z4 and Z5 in pure water demonstrate random coiling at 202 nm. However, when Z5 and Z4 are mixed, this pair of charge-complementary peptides undergo co-assembly in the absence of salt, forming a β-sheet conformation characterized by a pronounced negative peak at 216 nm, without the shoulder (Figure 4f). The electrostatic repulsion between Z5 or Z4 molecules resists peptide self-association unless diminished by the addition of salt. The designed sequences of Z5 and Z4, with opposite but equal overall charges and matched positions of charged residues, facilitate co-association and fibrillization based on electrostatic attraction between oppositely charged molecules when mixed in pure water. In other words, co-assembly does not rely on the salt-shielding effect, as the co-association between Z4 and Z5 molecules fundamentally diminishes the electrostatic repulsion. Accordingly, Z5 and Z4 co-assemble into nanofibers in both pure water and PBS (Figure 4c,d). However, the salt-shielding effect in PBS also diminishes the electrostatic attraction between Z5 and Z4 molecules, extends the distance of hydrogen bonds, and induces a slight rotation of the β-sheet structure along the fiber axis [32]. This results in more disordered structural arrangements, as evidenced by the presence of a minor shoulder at approximately 206 nm in the CD spectrum (Figure 4f). The formation of the Z5 and Z4 co-assembled hydrogel is corroborated by monitoring turbidity (Figure 4g). The observed variation in turbidity correlates with the conversion of the secondary structure and nanofiber formation.

**Figure 5:**
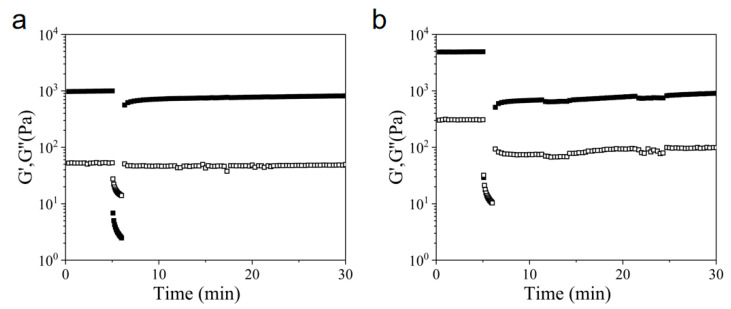
Rheological time sweeps with alternating low–high strains of the composite hydrogels with 1 wt% Z5 and 1 wt% nanoclay in DI water (**a**) and in NS (**b**).

Rheological analysis indicates that the G′ of the co-assembled hydrogel in pure water is nearly equivalent to that of Z4 or Z5 in PBS (Figure 4h) and increases linearly with peptide concentration (Figure S2). In addition, the co-assembled hydrogels in water, PBS, or NS were observed to display brittleness and lacked the capacity to recover their structural integrity following destruction. As discussed above, Z5 hydrogels in 0.5× PBS or NS displayed remarkable self-healing behaviors as a result of the balance between the electrostatic repulsion among the positively charged peptide nanofibers, moderated by the salt shielding effect, and the attractive interactions, including physical entanglement, hydrogen bonding, and hydrophobic interactions. The self-healing capacity of the hydrogel is speculated to be contingent upon the presence of balanced electrostatic repulsion, which is negated in Z5 and Z4 co-assembled hydrogels, irrespective of the concentration of salt employed. In contrast, when mixed with Z5, the nanoclay, also carrying negative charges, behaves as a polyvalent anion and may provide a moderate salt shielding effect and ionic cross-linking for Z5 nanofibers, thereby resulting in a self-healing composite hydrogel (Figure 5a). As observed with Z5 hydrogels in 2× or 4× NS, the addition of NS to the nanoclay and Z5 composite hydrogel enhances the salt shielding effect, thereby increasing G′ but impeding the recovery of the initial G′ post-destruction (Figure 5b). Consequently, incorporating moderate electrostatic repulsion may be crucial for developing injectable SAP hydrogels under physiological conditions.

### 2.3. Printing Capacity of Z5 Hydrogels

**Figure 6:**
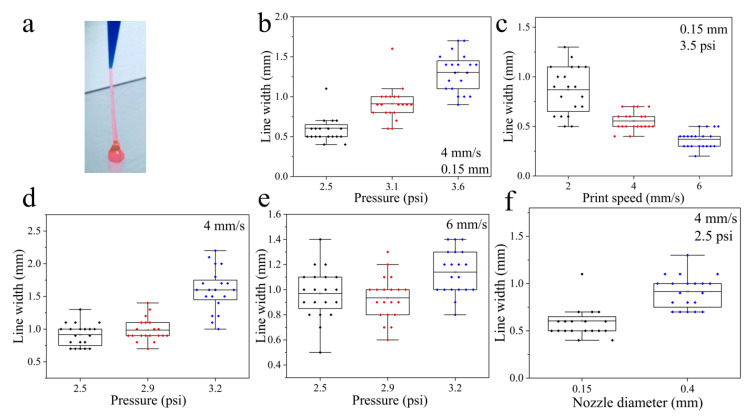
The printing performance of Z5 hydrogel at 2 wt% in NS. (**a**) The extrusion of the Z5 hydrogel from a nozzle with a diameter of 0.4 mm. The effect of air pressure on the printed line width at a printing speed of 4 mm/s with a 0.15 mm nozzle (**b**) and a 0.4 mm nozzle (**d**). (**c**) The effect of printing speed on the printed line width with air pressure at 3.5 psi and a 0.15 mm nozzle. (**e**) The effect of air pressure on the printed line width at a printing speed of 6 mm/s with a 0.4 mm nozzle. (**f**) The effect of nozzle diameter on the printed line width at a printing speed of 4 mm/s with air pressure at 2.5 psi.

**Figure 7:**
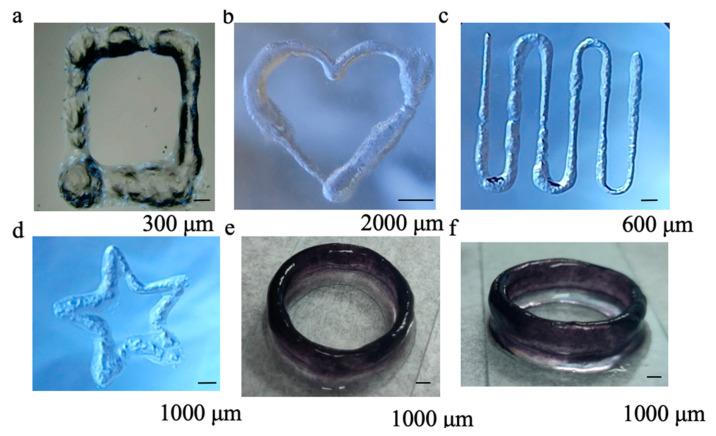
The printed 2D graphics (**a**–**d**) and 3D circle rings (**e**,**f**) using Z5 hydrogels at 2 wt% in NS with a needle diameter of 0.15 mm.

Given its outstanding self-healing (Figure 3f) and extrusion (Figure 6a) properties, the Z5 hydrogel in NS was examined with a commercial 3D bioprinter to assess its printing capacity by creating one-dimensional (1D) lines, two-dimensional (2D) graphics, and three-dimensional (3D) circle rings. The influence of air pressure and printing speed on the generation of 1D lines was investigated using nozzles with diameters of 0.15 mm and 0.4 mm. The findings indicate that increasing the air pressure and reducing the printing speed generally resulted in increased line widths, aligning with theoretical expectations (Figure 6a,b). The optimal parameters were identified as those producing a line width closest to the diameter of the nozzle, which were then applied to print 2D graphics. The uniformity of the printed line, a key indicator of printability, was evaluated visually. Although the figure’s outline was clearly discernible (Figure 7), unevenness at the edges was noted, which is a common issue observed in current printable SAP hydrogels. In addition, the experiment was extended to encompass the printing of 3D rings (Figure 7e,f).

At present, most SAP hydrogels exhibit poor printing performance. The printing of SAP hydrogels alone results in the formation of irregular and discontinuous print lines, as well as needle obstruction [41]. Hepi Susapto and colleagues successfully printed 3D structures using specially designed SAP hydrogels, but their printing device was complex and the printed structures lacked clarity [42]. Their SAP hydrogels under physiological conditions displayed brittleness, a common characteristic observed in most SAP hydrogels. Extrusion printing was achieved by introducing the salt solution to the peptide pregel immediately prior to its exit from the printing nozzle, using a specially designed printing device. The SAP hydrogel designed by Bella Raphael et al. exhibited favorable printing properties, but their hydrogels were not composed of natural amino acids [43]. Quan Li et al. reported the development of a self-healing peptide hydrogel for the bioprinting of 3D spheroids of human-induced pluripotent stem cells with acceptable printability [44]. The diameter of the printed filaments was about seven times larger than that of the nozzles, suggesting that the hydrogel was unable to maintain its structure upon exiting the nozzle. This phenomenon may be attributed to the relatively slow self-healing kinetics of the hydrogel, which required over a minute to regain its strength after the release of shear force. For comparison, the Z5 hydrogel reported here can recover its initial strength in seconds after the force is released. Accordingly, Z5 hydrogels in NS demonstrate satisfactory printing capabilities compared to the limited number of printable SAP hydrogels currently available.

### 2.4. Z5–Gelatin Composite Hydrogels

**Figure 8:**
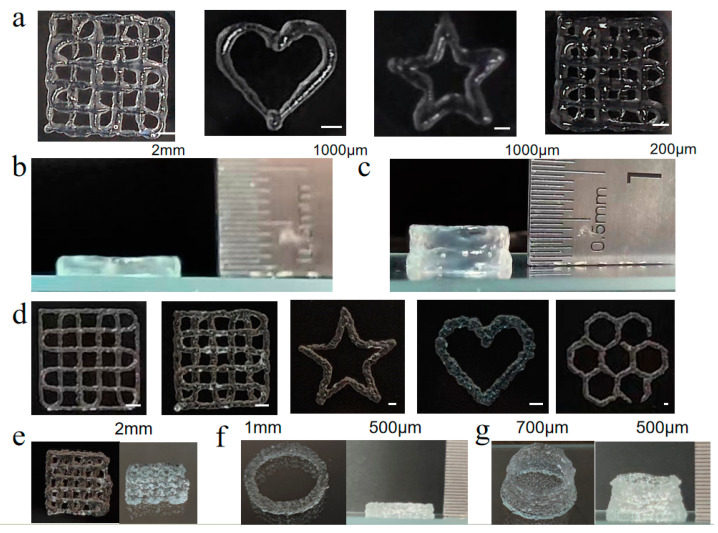
The printing performance of Z5–gelatin composite hydrogels. The printed 2D graphics (**a**), 10 layers of 3D circle rings (**b**), and 30 layers of 3D circle rings (**c**) using the composite hydrogels with 1.5 wt% Z5 and 5 wt% gelatin. The printed 2D graphics (**d**), 4 layers of 3D grid structure (**e**), 10 layers of 3D circle rings (**f**), and 30 layers of 3D circle rings (**g**) using the composite hydrogels with 1.5 wt% Z5 and 7 wt% gelatin. The diameter of the printing nozzle was 0.15 mm.

The intricate interweaving of nanofibers within Z5 hydrogels in NS impairs their printing capacity, leading to uneven hydrogel extrusion. This observation suggests that incorporating type A gelatin, which is positively charged under physiological conditions, into Z5 hydrogels may be a viable method to improve printing quality by regulating the electrostatic interactions among nanofibers. Composite hydrogels combining 15 mg/mL Z5 and either 50 mg/mL or 70 mg/mL gelatin were evaluated for their printing capacities (Figure 8). The results demonstrated that the incorporation of gelatin led to an improvement in the printing quality of Z5 hydrogels. In particular, the composite hydrogel containing 50 mg/mL gelatin was successfully printed in the form of 3D circle rings comprising 10 and 30 layers, with a height of 2 mm and 4 mm, respectively (Figure 8b,c), and with interlayer fusion. At concentrations of 70 mg/mL or above, gelatin itself can be printed at room temperature, but it will convert to a liquid at 37 °C. Therefore, the composite hydrogel containing 70 mg/mL gelatin exhibited analogous behavior to that of a gelatin hydrogel, leading to issues such as interlayer separation and fragmentation in the printed rings (Figure 8f,g). Nonetheless, both types of composite hydrogels are capable of sustaining their structure at a temperature of 37 °C, due to the formation of the Z5 hydrogel network and the interactions between Z5 and gelatin nanofibers.

## 3. Conclusions

In this work, a peptide designated Z5, which is characterized by alternating hydrophilic and hydrophobic amino acids and possesses two positive charges, was developed to investigate the influence of electrostatic interactions on peptide self-assembly. The peptide is capable of self-assembling into nanofibers with a β-sheet structure and forming hydrogels under specific conditions, such as in PBS and NS environments. This research encompassed a comprehensive analysis of the assemblies, including rheological characterization, electron microscopy, CD spectroscopy, and printing properties. In PBS, Z5 forms brittle hydrogels with densely distributed nanofiber bundles due to the strong salt shielding effect and ionic cross-linking. Conversely, Z5 hydrogels composed of nanofiber networks without bundling in NS exhibit exceptional self-healing properties. As Z5 hydrogels in NS can recover their initial strength seconds after the force is released, they demonstrate satisfactory printing capabilities when compared with the limited number of printable SAP hydrogels currently available. Additionally, elevated ionic concentrations in 2× NaCl and 4× NaCl environments can also result in a strong charge shielding effect, enhancing nanofiber bundling due to hydrophobic interactions, but negating self-healing capabilities. Thus, it can be concluded that the self-healing capacity of Z5 hydrogels is contingent upon the presence of balanced electrostatic repulsion. However, when a negatively charged peptide, Z4, is mixed with Z5 to form a co-assembled hydrogel, the electrostatic repulsion among nanofibers is eliminated regardless of the concentration of salt used. In contrast, the nanoclay, which also carries negative charges, behaves as the polyvalent anion and provides a moderate salt shielding effect and ionic cross-linking for Z5 nanofibers when mixed with Z5, thereby resulting in a self-healing composite hydrogel. Furthermore, the incorporation of positively charged type A gelatin into Z5 hydrogels improves the printing quality of the hydrogels by regulating the electrostatic interactions among nanofibers. Overall, the findings highlight the pivotal role of the solution environment in regulating the electrostatic interactions among peptide molecules and nanofibers, thereby affecting the self-assembly of the peptide. Introducing charged materials proves effective in modulating the electrostatic interactions that influence the internal self-assembling structures and the hydrogel properties. This research offers novel insights into the design of SAP hydrogels, emphasizing the critical influence of electrostatic interactions in determining the self-assembly behaviors of peptides.

## 4. Materials and Methods

### 4.1. Materials

Desalted Z5 (>98% purity) and Z4 (>98% purity) were purchased from Shanghai Top-Peptide Biotechnology Co., Ltd.(Shanghai, China) and were used without further purification. NaCl was purchased from Shanghai Aladdin Biochemical Technology Co., Ltd. (Shanghai, China). PBS was purchased from Dalian Meilun Biotechnology Co., Ltd. (Dalian, China). Nanoclay (LAPONITE-XLG) was purchased from Beijing Yiwei Tehua Technology Development Co., Ltd. (Beijing, China). Gelatin was purchased from Shanghai Yuanye Bio-Technology Co., Ltd. (Shanghai, China).

### 4.2. Hydrogel Preparation

The peptide powder was dissolved in deionized (DI) water with the assistance of sonication. A total of 20 mg/mL of Z5 hydrogel in PBS or NS was prepared by adding one volume of 10× PBS or 10× NS to nine volumes of the Z5 solution in DI water. The 20 mg/mL Z4 hydrogel in PBS was prepared by adding one volume of 10× PBS into nine volumes of Z4 solution in DI water. The Z5 and Z4 co-assembling hydrogel containing 5 mg/mL of Z5 and 5 mg/mL of Z4 was prepared by adding one volume of 10× NS into nine volumes of the peptide mixture in DI water. The Z5–nanoclay composite hydrogel containing 10 mg/mL of Z5 and 10 mg/mL of nanoclay was prepared by adding one volume of 10× NS to nine volumes of the mixture of Z5 and nanoclay in DI water. The Z5–gelatin composite hydrogels containing 15 mg/mL of Z5 and 50 mg/mL or 70 mg/mL of gelatin were prepared by adding one volume of 10× NS to nine volumes of the mixture of Z5 and gelatin in DI water.

### 4.3. Material Charaterization

The SEM samples were prepared using an ethanol gradient dewatering process for the peptide hydrogel. Typically, 20 mg/mL of Z5 hydrogels in PBS or NS were immersed in a gradually increasing ethanol concentration for the purpose of dehydration. Finally, the dehydrated hydrogel was soaked in a 100% ethanol solution and was subsequently dried in a critical point dryer (HCPD-15, Joel Hi-Tech Co., Ltd., Dalian, China). Prior to imaging, the dried hydrogel was sputtered with gold. The structures of the dried peptide hydrogels were characterized using a SEM (FEI NOVA NanoSEM 450, Hillsboro, OR, USA). Diluted peptide solutions in PBS or NS were placed on the carbon film on the copper grid, after which they were stained with 1% uranium acetate. The pH values of 100 μM peptide solutions in DI water, PBS, and NS were measured to be about 7.2, 7.3, and 6.4, respectively. Images were obtained using a TEM (FEI Tecnai TF20, Hillsboro, OR, USA) operated at 200 kV. The CD spectra were collected using a spectropolarimeter (Jasco J-720, Tokyo, Japan) with Starna quartz cuvettes (1 mm or 0.1 mm path length) at room temperature. The rheological properties of the Z5 hydrogels were investigated using a rheometer (Anton Paar MCR302, Graz, Austria) equipped with a 25 mm parallel plate system at 25 °C. The rheological test was initiated by depositing nine volumes of the peptide solution in DI water at the center of the lower plate. This was followed by the addition of one volume of 10× PBS or 10× NS to trigger the gelation process. Subsequently, the measuring geometry (the upper plate) was pressed onto the peptide solution immediately after the salt solution was added. The distance between the plates was set to 500 μm for all tests. The gelation process was monitored by conducting a time scan at a constant angular frequency of 6 rad/s and 0.1% strain immediately after the upper plate was positioned. When performing the frequency scans, the peptide solution was incubated for approximately 90 min after the upper plate was positioned to ensure adequate on-site gelation. A frequency scan was conducted at 0.1% strain within the linear viscoelastic range, spanning from 100 rad/s to 0.1 rad/s. To measure the self-healing properties, step strain analysis was performed at a constant angular frequency of 6 rad/s with strains of 0.1 and 100%. Prior to the initiation of the step strain analysis, the peptide solution was subjected to an incubation period of approximately 90 min after the upper plate was positioned. It was observed that the outcomes remained unaltered irrespective of whether the frequency scan was conducted prior to the step strain analysis. Throughout all rheological tests, the test chamber was maintained in a water-sealed state. For the turbidity analysis, an aliquot of 10 µL of 10× PBS or NS was added to 90 µL of the peptide solution (10 mg/mL) in a 96-well plate (Nunc, Copenhagen, Denmark). The absorption kinetics were then monitored at 313 nm using a plate reader (Tecan Infinite 200 Pro, Männedorf, Switzerland) at room temperature. The hydrogels were printed using a bioprinter (Allevi 2, Allevi Inc., Philadelphia, PA, USA).

## Data Availability

The original contributions presented in this study are included in the article/Supplementary Material. Further inquiries can be directed to the corresponding authors.

## Supplementary Materials

The following supporting information can be downloaded at: https://www.mdpi.com/article/10.3390/gels11010080/s1, Figure S1: Rheological time sweeps monitoring the gelation process (a), frequency sweeps (b), and time sweeps with alternating low–high strains (c) of Z5 hydrogels at different concentrations in NS; Figure S2: Rheological frequency sweeps of the Z5–Z4 co-assembled hydrogels with different concentrations.
