# Supplementary material for: Influence of Electrostatic Interactions on the Self-Assembly of Charged Peptides
Source: Gels. 2025 Jan 20;11(1):80. doi: 10.3390/gels11010080 (PMC11765140; doi:10.3390/gels11010080)
Supplement: Supplementary file 1 [file gels-11-00080-s001.zip › gels-3384620-supplementary.pdf]

gels

Supporting information

Influence of Electrostatic Interactions on the Self-Assembly of Charged Peptides

Xue Sun [1,2], Bolan Wu [2], Na Li [2], Bo Liu [2], Shijun Li [1], Liang Ma [1,*] and Hangyu Zhang [1,2,*]

1 Faculty of Medicine, Dalian University of Technology, Dalian 116033, China
2 School of Biomedical Engineering, Liaoning Key Lab of Integrated Circuit and Biomedical Electronic System, Dalian University of Technology, Dalian 116024, China
* Correspondence: mysheng@yeah.net (L.M.); hangyuz@dlut.edu.cn (H.Z.)

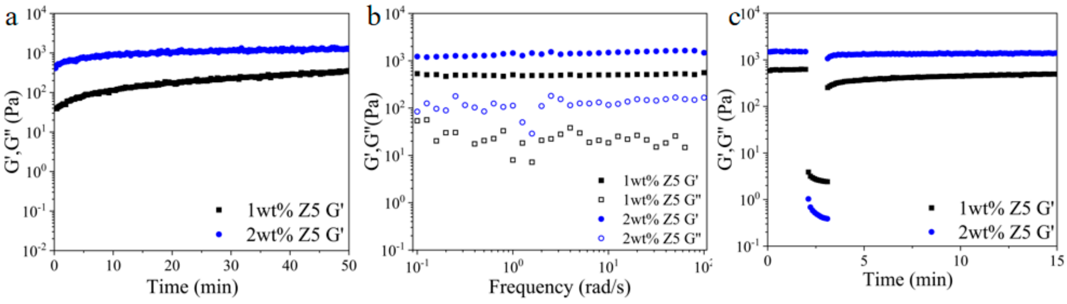

Figure S1. Rheological time sweeps monitering gelation process (a), frequency sweeps (b), and time sweeps with alternative low-high strains (c) of Z5 hydrogels at different concentrations in NS.

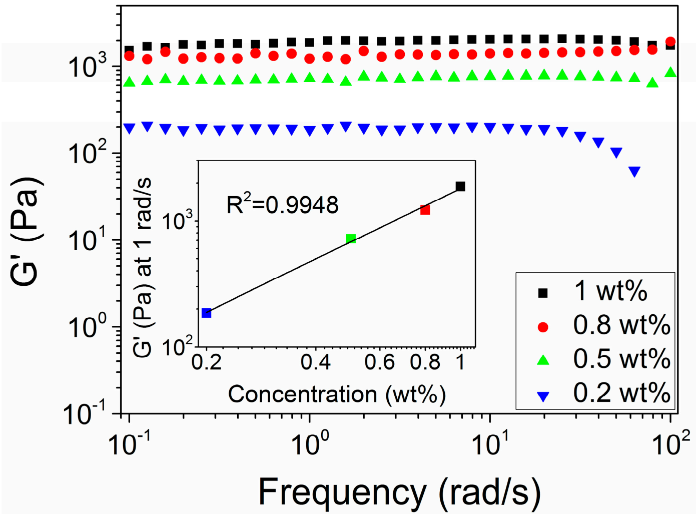

Figure S2. Reological frequency sweeps of the Z5-Z4 co-assembled hydrogels with different concentrations.
